# Restructuring the gut microbiota in obesity: molecular mechanisms linking dysbiosis to systemic inflammation and therapeutic opportunities

**DOI:** 10.3389/fphys.2026.1902756

**Published:** 2026-09-04

**Authors:** Qiqi Le

**Affiliations:** Songnan Town Community Health Service Center, Baoshan District, Shanghai, China

**Keywords:** dysbiosis, gut microbiota, metabolic inflammation, microbial therapeutics, obesity

## Abstract

Obesity is characterized by a chronic low-grade inflammatory state that contributes to insulin resistance, type 2 diabetes, and metabolic syndrome. The gut microbiota has emerged as a critical mediator of this inflammatory process through multiple interconnected mechanisms including metabolic endotoxemia, short-chain fatty acid dysregulation, and intestinal barrier dysfunction. This review synthesizes current evidence on the structural and functional alterations of the gut microbiome in obesity, examines the mechanistic pathways linking dysbiosis to systemic inflammation, and critically evaluates therapeutic strategies aimed at restructuring the obese gut microbial community. We focus on three major intervention approaches: fecal microbiota transplantation, probiotic and prebiotic supplementation, and next-generation targeted microbial therapies. Analysis of clinical and preclinical studies reveals that successful microbial restructuring requires not only compositional shifts but also functional restoration of microbial metabolite production, particularly short-chain fatty acids. The evidence supports a model wherein obesity-associated dysbiosis perpetuates chronic inflammation through increased lipopolysaccharide translocation, reduced butyrate production, and compromised intestinal barrier integrity. Restoring microbial eubiosis through targeted interventions offers a promising avenue for resolving chronic low-grade inflammation and improving metabolic health outcomes in obese individuals.

## 1 Introduction

Obesity represents one of the most pressing global health challenges of the 21st century, with prevalence rates continuing to rise across both developed and developing nations (Blüher, 2019; Ahmed and Mohammed, 2025). This epidemic is not merely a cosmetic concern but a profound medical crisis that strains healthcare systems worldwide. Beyond its association with increased adiposity, obesity is fundamentally characterized by a chronic low-grade inflammatory state that underlies the development of insulin resistance, type 2 diabetes mellitus, cardiovascular disease, and certain malignancies (Saltiel and Olefsky, 2017; Khanna et al., 2022). This inflammatory condition, often termed metabolic inflammation or meta-inflammation, differs significantly from acute inflammatory responses. Unlike acute inflammation, which is a localized and temporary defense mechanism against injury or infection, meta-inflammation is persistent, low-grade, and systemic in nature, silently damaging tissues over years or decades (Tilg and Moschen, 2006).

The recognition that the gut microbiota functions as an integral component of host physiology has fundamentally transformed our understanding of metabolic disease pathogenesis (Qin et al., 2010; Cheng et al., 2022). No longer viewed merely as passive passengers in the digestive tract, these microorganisms are now understood to be active participants in human biology. The human gastrointestinal tract harbors approximately 10^14^ microorganisms, collectively encoding over 100-fold more genes than the human genome (Thursby and Juge, 2017). This vast genetic reservoir, often referred to as the “second genome,” provides metabolic capabilities that humans lack. This complex microbial ecosystem participates in nutrient metabolism, immune system development, pathogen exclusion, and maintenance of intestinal barrier integrity (Hou et al., 2022). Consequently, the health of the host is inextricably linked to the health of this microbial community. Disruption of this symbiotic relationship, termed dysbiosis, has been consistently associated with obesity and its metabolic complications across numerous human and animal studies (Scheithauer et al., 2020; Tilg et al., 2020).

The gut microbiota influences host metabolic health through multiple interconnected pathways. Dysbiotic microbial communities in obesity exhibit altered energy harvesting capacity, modified bile acid metabolism, impaired short-chain fatty acid (SCFA) production, and increased generation of pro-inflammatory bacterial components (Cani et al., 2012; Amabebe et al., 2020). For instance, an increased Firmicutes-to-Bacteroidetes ratio has been frequently observed in obese individuals, suggesting a microbiome that is more efficient at extracting energy from dietary polysaccharides, thereby contributing to weight gain (Magne et al., 2020). However, contemporary evidence indicates that metabolic dysregulation is driven by deeper functional alterations such as disrupted SCFA profiles and bile acid metabolism—rather than simplistic phylum-level shifts. Furthermore, these alterations collectively contribute to the chronic inflammatory state characteristic of obesity through mechanisms including metabolic endotoxemia, intestinal barrier dysfunction, and activation of pattern recognition receptors in metabolic tissues (Chassaing and Gewirtz, 2014). Specifically, when the intestinal barrier becomes permeable, often described as leaky gut bacterial lipopolysaccharides (LPS) translocate into the circulation. This triggers an immune response via Toll-like receptor 4 (TLR4) activation, leading to the release of pro-inflammatory cytokines such as TNF-alpha and IL-6, which interfere with insulin signaling pathways (Page et al., 2022).

This review provides a comprehensive synthesis of current evidence on the relationship between gut microbial dysbiosis and chronic low-grade inflammation in obesity. We examine the structural and functional characteristics of the obese microbiome, elucidate the mechanistic pathways linking dysbiosis to systemic inflammation, and critically evaluate therapeutic strategies aimed at restructuring the gut microbial community to restore metabolic health. Understanding these mechanisms is crucial for moving beyond symptomatic treatment to addressing the root causes of metabolic dysfunction.

Our analysis focuses on three major intervention categories: fecal microbiota transplantation (FMT), probiotic and prebiotic supplementation, and emerging next-generation microbial therapeutics. FMT involves the transfer of stool from a healthy donor to a recipient, aiming to restore a balanced microbial ecosystem. While initially developed for treating Clostridioides difficile infections, recent trials have explored its potential in metabolic diseases, showing promise in improving insulin sensitivity, although long-term efficacy and safety profiles remain under investigation. Probiotic and prebiotic supplementation represents a more accessible approach. Probiotics introduce beneficial live bacteria, while prebiotics provide the fiber necessary to feed existing beneficial microbes (Ji et al., 2023). Recent studies suggest that specific strains, such as Akkermansia muciniphila, may strengthen the gut barrier and reduce inflammation (Mo et al., 2024). Finally, next-generation microbial therapeutics, including engineered bacteria and postbiotics, offer precise tools to modulate specific metabolic pathways. These advanced therapies aim to target the underlying inflammatory mechanisms directly, offering hope for personalized medicine approaches in obesity management. By integrating these diverse therapeutic strategies, we can potentially reverse the dysbiotic state and mitigate the severe health consequences associated with obesity-related inflammation.

### 1.1 Literature search strategy and selection criteria

To ensure an objective synthesis of current evidence, a structured literature search was conducted across major electronic databases, including PubMed/MEDLINE, Web of Science, Scopus, and Embase, covering publications up to early 2026. The search strategy utilized combinations of MeSH terms and free-text keywords, including: (“obesity” OR “metabolic syndrome”) AND (“gut microbiota” OR “gut microbiome” OR “dysbiosis”) AND (“metabolic endotoxemia” OR “LPS” OR “TLR4”) AND (“short-chain fatty acids” OR “butyrate”) AND (“fecal microbiota transplantation” OR “probiotics” OR “prebiotics” OR “Akkermansia muciniphila”).

Inclusion criteria: (1) Peer-reviewed original research articles and high-impact reviews; (2) Preclinical (*in vitro* and animal) and clinical studies investigating the biological mechanisms connecting gut dysbiosis to metabolic inflammation; and (3) Studies evaluating microbiome-targeted interventions (FMT, probiotics, prebiotics, and postbiotics) in the context of obesity and metabolic dysregulation.

Exclusion criteria: (1) Non-English publications; (2) Conference abstracts, editorials, and commentary articles lacking empirical data; and (3) Studies lacking clear mechanistic or therapeutic relevance to obesity-associated inflammation.

Articles were independently screened by the authors based on title, abstract, and full-text eligibility to guarantee the quality, scientific rigor, and relevance of the synthesized literature.

## 2 The obese gut microbiome: structural and functional alterations

Human and animal studies have consistently demonstrated substantial alterations in gut microbiota composition associated with obesity (Ley et al., 2006). These findings have shifted the paradigm from viewing obesity solely as a result of caloric imbalance to understanding it as a complex interplay between host genetics, diet, and microbial ecology. Early landmark studies identified a characteristic increase in the relative abundance Firmicutes and a corresponding decrease in Bacteroidetes in obese individuals compared to lean controls (Magne et al., 2020). This specific shift was initially thought to be a universal hallmark of obesity, suggesting that the obese microbiome is more efficient at harvesting energy from the diet (Davis, 2016). The Firmicutes-to-Bacteroidetes ratio has subsequently been proposed as a potential biomarker of obesity-associated dysbiosis, though this relationship shows considerable inter-individual variability and is influenced by dietary factors (Ahmed et al., 2024; Karačić et al., 2024). Recent large-scale analyses suggest that while this ratio is significant in many contexts, it is not a definitive diagnostic tool due to the high diversity of human microbial communities and the influence of confounding variables such as age, geography, and medication use (Karačić et al., 2024). Consequently, current consensus shifts away from broad phylum-level ratios, emphasizing that obesity-associated dysbiosis encompasses much finer alterations across lower taxonomic and functional levels.

Moving beyond high-level phylum shifts, a more nuanced picture of microbial imbalance emerges at the genus and species levels. Reduced abundance of beneficial genera including Bifidobacterium, Lactobacillus, and Akkermansia has been consistently observed in obese cohorts (Fruge et al., 2020; Michael et al., 2020). Akkermansia muciniphila, in particular, has garnered significant attention for its role in maintaining the mucus layer of the gut lining; its depletion is strongly linked to increased intestinal permeability and inflammation (Mo et al., 2024). Conversely, obesity is associated with expansion of pro-inflammatory taxa including members of the Enterobacteriaceae family and certain species within the genus Prevotella (Larsen, 2017). These opportunistic pathogens can thrive in an environment altered by high-fat and high-sugar diets, further exacerbating metabolic distress. In preclinical models, germ-free mice colonized with microbiota from obese donors exhibit significantly greater fat mass accumulation than those receiving microbiota from lean donors (Kulecka et al., 2016). These seminal transfer experiments provided proof-of-concept evidence that dysbiotic microbial communities can actively drive weight gain independent of host genetic background, establishing causality in animal models, whereas evidence in humans remains largely correlational.

The functional implications of obesity-associated dysbiosis extend far beyond compositional changes, affecting the very metabolic machinery of the host. Metagenomic and metabolomic analyses have revealed substantial alterations in microbial gene expression and metabolic output in obese individuals (Hu et al., 2024). Key functional alterations include a reprogramming of how energy is processed and stored. The obese microbiome exhibits enhanced capacity for energy extraction from dietary components (Turnbaugh et al., 2006). Dysbiotic communities demonstrate increased expression of enzymes involved in carbohydrate fermentation and short-chain fatty acid production, paradoxically coupled with alterations in SCFA ratios that favor metabolic dysfunction (Onyango et al., 2021; Winter and Bäumler, 2023). Additionally, obese microbiota suppresses expression of fasting-induced adipocyte factor (FIAF), leading to increased lipoprotein lipase activity and enhanced triglyceride storage in adipocytes (Bäckhed et al., 2004). By inhibiting FIAF, the microbiota effectively removes a brake on fat storage, promoting the accumulation of lipid reserves even in the absence of excessive caloric intake (Bäckhed et al., 2004).

SCFAs primarily acetate, propionate, and butyrate represent critical microbial metabolites with profound effects on host metabolism and inflammation (Sun et al., 2017). A critical distinction must be made between fecal SCFA concentrations and actual tissue exposure; elevated fecal SCFA levels observed in some obese cohorts do not necessarily reflect increased colonic production, but may instead indicate impaired gut absorption, altered colonic transit time, or reduced mucosal utilization (De la Cuesta-Zuluaga et al., 2018; Yu et al., 2025). Furthermore, while acetate and propionate can serve as lipogenic substrates in the liver via portal vein transit, butyrate is primarily consumed by colonocytes to maintain epithelial energy dynamics and tight-junction integrity (Bach Knudsen et al., 2018). In obesity, a shift in SCFA balance toward relatively lower butyrate availability, together with impaired mucosal transport/utilization, may compromise intestinal barrier function (Coppola et al., 2021; Mayorga-Ramos et al., 2022; Ehtiati et al., 2025). This compromised barrier permits the translocation of bacterial endotoxins into the circulation, initiating metabolic endotoxemia and triggering systemic immune responses that impair peripheral insulin signaling (Paray et al., 2020).

The gut microbiota extensively metabolizes host-derived bile acids, converting primary bile acids to secondary bile acids through deconjugation and dehydroxylation reactions (Winston and Theriot, 2020). These microbial bile acid transformations significantly alter the signaling properties of these molecules, affecting host metabolism through activation of farnesoid X receptor (FXR) and G protein-coupled bile acid receptor 1 (TGR5) (Pathak et al., 2018). FXR activation regulates glucose and lipid metabolism, while TGR5 stimulates the release of glucagon-like peptide-1 (GLP-1), which enhances insulin secretion and satiety. Obesity-associated dysbiosis disrupts normal bile acid metabolism, contributing to metabolic dysfunction and hepatic steatosis (Kim and Fang, 2018; Zhu et al., 2024). An altered bile acid pool can lead to impaired FXR signaling, resulting in decreased energy expenditure and increased lipogenesis in the liver. Furthermore, the disruption of TGR5 signaling may reduce GLP-1 levels, diminishing the feeling of fullness and promoting overeating (Chiang and Ferrell, 2022; Krug et al., 2025). Thus, the microbiota acts as a central regulator of metabolic homeostasis, influencing everything from energy harvest to hormonal signaling and immune response. Understanding these intricate mechanisms provides a foundation for developing targeted therapies that restore microbial balance and improve metabolic health. The characteristic taxonomic alterations and their corresponding functional impacts within the obese gut microbial community are summarized in **Table 1**.

**Table 1:** Key taxonomic shifts and their pathophysiological roles in the obese gut microbiome.

Microbial taxon	Change	Functional alteration	Impact on host inflammation	Ref
Akkermansia muciniphila	↓	Thinning of the colonic mucus layer	Impaired epithelial barrier integrity; increased translocation of luminal antigens (Leaky Gut)	(Abuqwider et al., 2021)
Bifidobacterium spp.	↓	Reduced competitive exclusion	Overgrowth of opportunistic pathogens; ↑ metabolic endotoxemia	(Salazar et al., 2015)
Enterobacteriaceae	↑	Increased LPS reservoir	Systemic activation of TLR4/NF- κ B-mediated pro-inflammatory cascades	(Du et al., 2022)
Phylum-level shifts (Firmicutes/Bacteroidetes)	Variable (↑/↓)	Enhanced energy harvest in early studies; heavily confounded by dietary fiber and host factors	Suppression of FIAF; ↑ triglyceride storage in adipocytes (insufficient as a standalone biomarker for systemic meta-inflammation)	(Ahmed et al., 2024)
F. prausnitzii	↓	Reduced butyrate production	Loss of HDAC inhibition; disrupted Th17/Treg immune balance and local cytokine release	(Zhou et al., 2018)

## 3 Mechanisms linking gut dysbiosis to chronic low-grade inflammation

**Figure 1:**
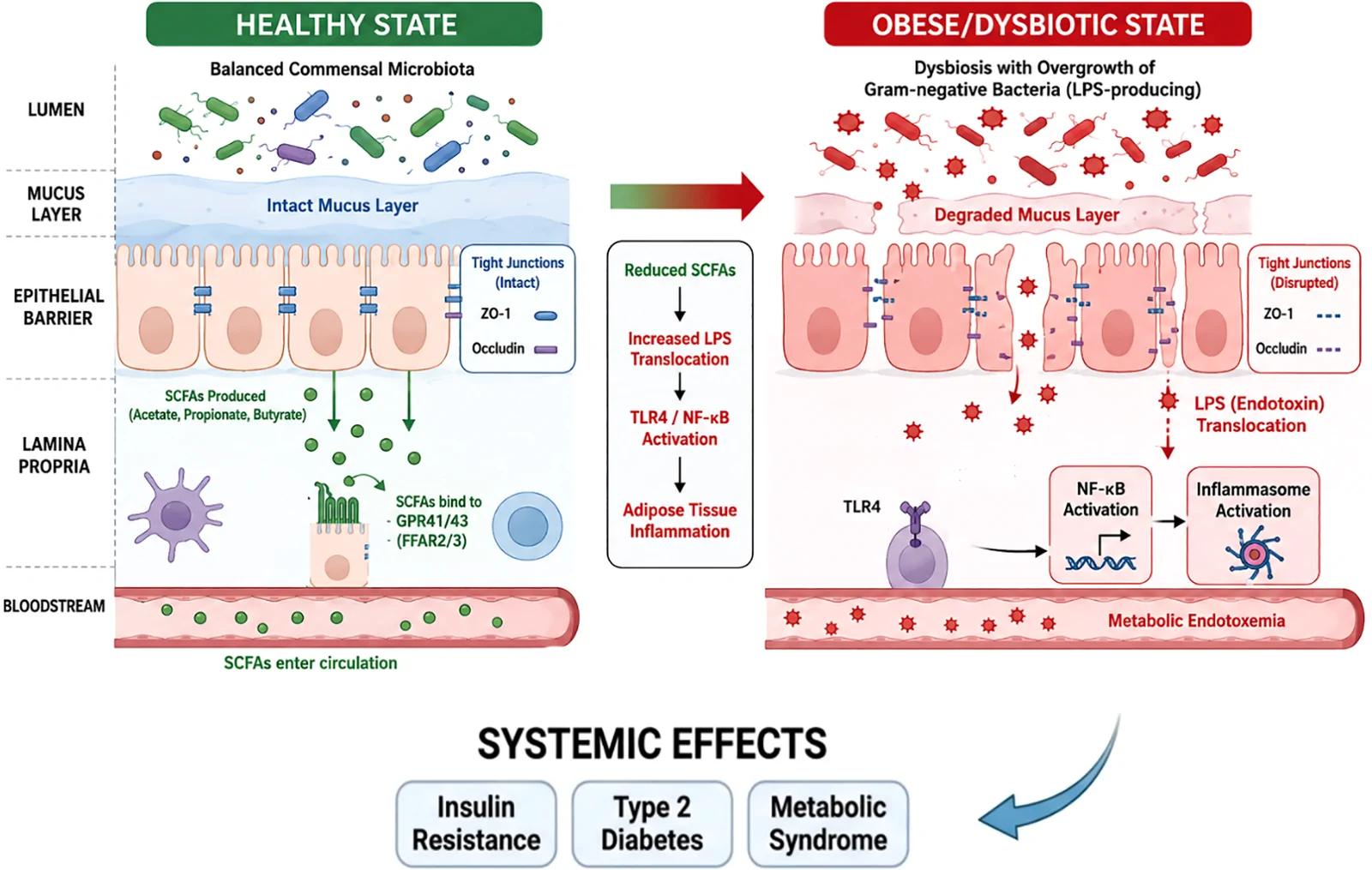
Mechanistic comparison of gut barrier integrity and downstream inflammatory signaling in healthy versus obese/dysbiotic states. (Left) Healthy State: An intact mucus layer and tight junctions (ZO-1, Occludin) preserve mucosal integrity. SCFAs translocate into the lamina propria and bind to transmembrane GPR41/43 (FFAR2/3) receptors on local mucosal cells, maintaining metabolic homeostasis before entering circulation.(Right) Obese/Dysbiotic State: Overgrowth of Gram-negative bacteria degrades the mucus layer and disrupts tight junction proteins, causing intestinal hyperpermeability (leaky gut). Translocated lipopolysaccharide (LPS) in the lamina propria binds to Toll-like receptor 4 (TLR4) on resident immune cells, triggering downstream NF-κB and inflammasome activation. (Bottom) Systemic Effects: Persistent metabolic endotoxemia and systemic low-grade inflammation drive insulin resistance, type 2 diabetes, and metabolic syndrome.(Figure created by the authors using vector graphic software). The symbol ↓ indicates a decrease, and ↑ indicates an increase in the respective parameters.

Among the diverse pathways connecting gut dysbiosis to systemic inflammation, metabolic endotoxemia represents perhaps the most well-characterized mechanism (Choroszy et al., 2022). In obese individuals, compromised intestinal barrier integrity and heightened gut permeability facilitate the systemic translocation of bacterial lipopolysaccharide (LPS) from the intestinal lumen into circulation (Gummesson et al., 2011). This persistent, low-grade endotoxemia subsequently activates Toll-like receptor 4 (TLR4) on immune cells and metabolic tissues, triggering downstream pro-inflammatory signaling cascades that drive chronic tissue inflammation (Shi et al., 2006) (**Figure 1**).

The gut microbiota drives systemic metabolic dysfunction through multiple interconnected mechanisms that compromise host integrity. First, an obese dysbiotic profile is enriched in Gram-negative bacteria, expanding the intestinal reservoir of LPS (Du et al., 2022). In health, these populations are constrained by competitive exclusion, but their expansion in obesity elevates luminal endotoxin levels. Second, reduced SCFA production—particularly butyrate—impairs tight junction assembly, causing paracellular “leakiness” that facilitates systemic translocation of microbial products (Cani et al., 2009). Third, a decline in mucin-degrading species such as Akkermansia muciniphila thins the protective mucus barrier, exposing the intestinal epithelium to direct microbial contact and toxic byproducts (Liévin-Le Moal and Servin, 2006; Plovier et al., 2017).

Experimental models confirm that this systemic influx of endotoxin actively initiates obesity-associated inflammation. Low-dose LPS administration in mice replicates high-fat diet-induced insulin resistance and weight gain (Cani et al., 2007), while TLR4-deficient mice remain protected from these inflammatory deficits (Tsukumo et al., 2007). In humans, circulating LPS levels directly correlate with body mass index, waist circumference, and systemic inflammatory markers (e.g., C-reactive protein and IL-6) (Lajunen et al., 2008; Lassenius et al., 2011), establishing metabolic endotoxemia as an active driver—rather than a passive consequence—of metabolic dysfunction.

Beyond TLR4, other pattern recognition receptors contribute to dysbiosis-induced inflammation, creating a complex network of immune sensing. Nucleotide-binding oligomerization domain-like receptors (NLRs) and C-type lectin receptors detect microbial components and damaged cell products, activating inflammasome complexes (Mason et al., 2011; Mnich et al., 2020). These intracellular sensors act as alarm systems, triggering robust inflammatory responses when they detect signs of cellular stress or microbial invasion. The NLRP3 (NOD-, LRR- and pyrin domain-containing protein 3) inflammasome, in particular, has been implicated in obesity-associated inflammation through its processing of pro-interleukin-1β and pro-interleukin-18 to their active forms (Wani et al., 2021). Once activated, NLRP3 leads to the release of these potent pro-inflammatory cytokines, which further exacerbate insulin resistance and promote tissue damage in metabolic organs such as the liver and adipose tissue.

Interestingly, the relationship between inflammasome activation and metabolic health is complex and context-dependent. While excessive inflammasome activation promotes inflammation and insulin resistance, appropriate inflammasome function is necessary for maintaining intestinal homeostasis and preventing dysbiosis (Rathinam and Chan, 2018). For instance, inflammasome signaling helps regulate the composition of the gut microbiota and supports the integrity of the intestinal barrier. This dual role highlights the delicate balance required in modulating inflammatory pathways for therapeutic benefit (Rathinam and Chan, 2018). Therapeutic strategies must therefore aim to suppress pathological inflammation without compromising the protective immune functions that maintain microbial equilibrium.

Emerging evidence indicates that microbial metabolites can influence host gene expression through epigenetic mechanisms, adding another layer of complexity to the host-microbe interaction. SCFAs, particularly butyrate, function as histone deacetylase (HDAC) inhibitors, modulating chromatin structure and gene transcription (Wang et al., 2025). By inhibiting HDACs, butyrate prevents the removal of acetyl groups from histones, leading to a more open chromatin structure that facilitates gene transcription. Thus, butyrate promotes the expression of genes involved in anti-inflammatory responses, antioxidant defense, and intestinal barrier maintenance (Mathew et al., 2014). This epigenetic regulation provides a mechanism by which dietary fibers, via microbial fermentation, can exert long-lasting effects on host physiology.

In obesity, reduced butyrate production compromises these epigenetic regulatory mechanisms, contributing to sustained pro-inflammatory gene expression patterns (Nshanian et al., 2025). Without the regulatory influence of butyrate, genes promoting inflammation may remain actively transcribed, perpetuating the cycle of metabolic dysfunction. Additionally, alterations in microbial folate and methionine metabolism may affect host DNA methylation patterns, further influencing metabolic and inflammatory phenotypes (Hou and Zhao, 2021; Vaccaro and Naser, 2021). Since DNA methylation typically represses gene expression, changes in the availability of methyl donors derived from microbial metabolism can significantly alter the host’s genetic landscape, potentially predisposing individuals to metabolic disease (Vaccaro and Naser, 2021).

The gut microbiota exerts significant influence on adipose tissue inflammation, a hallmark of obesity that drives systemic metabolic dysfunction (Massier et al., 2020). Through circulating bacterial products, metabolites, and immune cell trafficking, dysbiotic microbiota promote the recruitment and activation of pro-inflammatory macrophages within adipose tissue (Caesar et al., 2015). In lean individuals, adipose tissue macrophages predominantly exhibit an anti-inflammatory M2 phenotype, which supports tissue remodeling and insulin sensitivity. However, in obesity, there is a dramatic shift toward a pro-inflammatory M1 phenotype (Hinojosa Vera et al., 2025). This transition from an anti-inflammatory M2 macrophage phenotype to a pro-inflammatory M1 phenotype drives the release of tumor necrosis factor-α (TNF-α), interleukin-6 (IL-6), and other cytokines that induce insulin resistance in adipocytes (Zatterale et al., 2019). These cytokines interfere with insulin signaling pathways, reducing glucose uptake and promoting lipolysis, which further elevates circulating free fatty acids and exacerbates metabolic stress in other organs such as the liver and muscle (**Figure 1**).

## 4 Therapeutic strategies for microbial community restructuring

**Figure 2:**
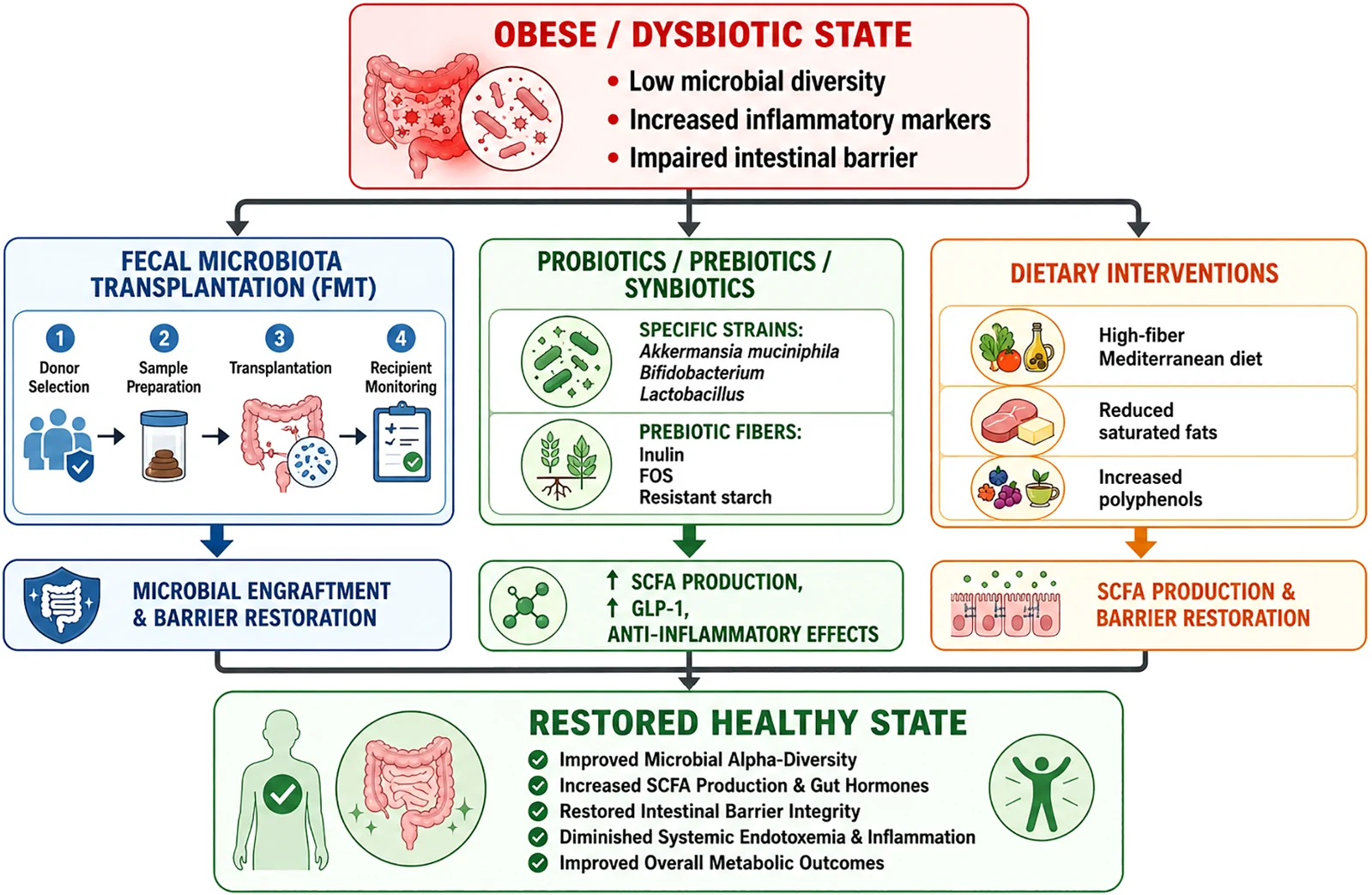
Schematic overview of therapeutic modalities for restructuring the obese gut microbiome. The central pathological state (Obese/Dysbiotic State) is defined by low microbial diversity, inflammation, and barrier dysfunction. Three targeted interventions FMT (via donor selection, sample preparation, transplantation, and monitoring), Probiotics, Prebiotics, and Synbiotics (utilizing strains like Akkermansia muciniphila, Bifidobacterium, and Lactobacillus, alongside fibers to boost SCFAs and GLP-1), and Dietary Interventions (high-fiber Mediterranean diet, low saturated fats, high polyphenols)—converge to achieve a Restored Healthy State. This state is characterized by enhanced alpha-diversity, restored barrier integrity, reduced endotoxemia, and may improve metabolic outcomes. (Figure created by the authors using vector graphic software).

Restructuring the obese gut microbial community to resolve chronic low-grade inflammation requires targeted interventions that address both compositional and functional aspects of dysbiosis. Current therapeutic strategies encompass three major categories: FMT, probiotic and prebiotic supplementation, and dietary modifications (**Figure 2**).

FMT represents the most direct and comprehensive approach to restructuring the gut microbiome, involving the transfer of processed fecal material from a carefully screened healthy donor to a recipient with the explicit goal of establishing a resilient, healthy microbial community (Uppala et al., 2026). This procedure effectively replaces the recipient’s dysbiotic ecosystem with a functional one, offering a reset for the gut environment. While FMT has revolutionized the treatment of recurrent Clostridioides difficile infection, achieving cure rates exceeding 90%, its application to metabolic disease remains investigational and complex (van Nood et al., 2013). The transition from treating an infectious disease to managing a chronic metabolic condition presents unique challenges, primarily due to the multifactorial nature of obesity and the intricate host-microbe interactions involved.

Several clinical trials have examined FMT efficacy in obesity and metabolic syndrome, yielding mixed results that highlight the nuances of this therapeutic modality (Qiu et al., 2023; Zecheng et al., 2023). Early pioneering studies demonstrated that FMT from lean donors could transiently improve insulin sensitivity in recipients with metabolic syndrome, with measurable effects lasting up to 6 weeks post-transplantation (Vrieze et al., 2012). These initial findings generated significant excitement, suggesting that transferring a lean microbiome could confer metabolic benefits. However, subsequent larger and more rigorous trials have shown more modest effects on body weight and broader metabolic parameters (Yu et al., 2020). The discrepancy between early promise and later modest outcomes suggests that while FMT can alter microbial composition, sustained metabolic improvement may require additional interventions or repeated treatments.

A critical factor influencing FMT outcomes is the donor metabolic phenotype. It is not merely the presence of specific bacteria but the functional capacity of the entire community that matters. Transplantation using microbiota from metabolically compromised donors may actually worsen recipient insulin sensitivity, highlighting the importance of careful donor selection (de Groot et al., 2020). This finding underscores the potential risks of FMT and the need for stringent screening protocols that go beyond infectious disease markers to include metabolic health assessments. Additionally, the durability of microbiome changes following FMT appears limited, with recipient microbiota often reverting toward baseline composition within months (Li et al., 2016). This resilience of the native microbiome, shaped by long-term dietary habits and host genetics, poses a significant barrier to long-term success. Therefore, FMT may be most effective when used as a primer to facilitate subsequent lifestyle or dietary changes, rather than as a standalone cure (Fassarella et al., 2021).

Successful FMT in metabolic disease likely requires both compositional and functional restoration of the microbiome. Mere colonization is insufficient; the transferred microbes must integrate into the existing ecological network and perform beneficial metabolic functions. Engraftment of beneficial taxa including Akkermansia, Faecalibacterium, and other butyrate-producing species has been associated with improved metabolic outcomes (Kootte et al., 2017). These key species contribute to barrier integrity and anti-inflammatory signaling. Additionally, FMT may restore SCFA production capacity, improve intestinal barrier function, and reduce metabolic endotoxemia, thereby addressing underlying drivers of inflammation (Kamlárová et al., 2025). To fully harness the clinical potential of FMT in metabolic disorders, several critical biological and operational parameters are actively being refined. Optimizing metabolic efficacy hinges on refined donor selection criteria extending beyond pathogen screening to comprehensive metabolic profiling—as well as tailoring the route of administration (e.g., targeted oral capsules versus colonoscopy) and strategically evaluating antibiotic pretreatment to prepare the host niche (Ianiro et al., 2022; Wu et al., 2025). Furthermore, promoting long-term microbial engraftment is recognized as a diet-dependent process, where adequate fermentable fiber intake helps maintain transferred beneficial taxa and prevents strain washout (Gogokhia et al., 2025). As the field navigates ongoing safety standards and evolving regulatory frameworks (such as FDA IND oversight), FMT holds strong promise—not necessarily as a single standalone cure, but as a powerful metabolic primer that can enhance responsiveness to subsequent lifestyle and targeted microbial interventions (Merrick et al., 2020). Despite these promising insights, observed clinical effects on weight management often remain modest, indicating that microbial therapeutics serve best as targeted adjuncts within broader lifestyle and dietary strategies.

Probiotics, defined as live microorganisms conferring health benefits when consumed in adequate amounts, represent a widely accessible and safer approach to modulating the gut microbiome (Gibson et al., 2017). Unlike FMT, which involves a complex community, probiotics typically involve single strains or defined consortia. Various probiotic strains have shown beneficial effects in obesity and metabolic disease, though results are often strain-specific and modest in magnitude (McFarland et al., 2018). This specificity implies that not all probiotics are created equal, and generalizations across species can be misleading.

Species within the genera Lactobacillus and Bifidobacterium have been most extensively studied due to their historical use in fermented foods and perceived safety. For instance, supplementation with Lactobacillus gasseri SBT2055 (200 g/day of fermented milk containing 10^8^ CFU/g) for 12 weeks in adults with obese tendencies (Body Mass Index 24.2–30.7 kg/m 2) demonstrated significant reductions in visceral fat area (4.6%), subcutaneous fat area (3.3%), body weight (1.4%/1.1 kg), and waist circumference (1.8%/1.7 cm), potentially through mechanisms involving reduced lipid absorption and altered energy expenditure (Kadooka et al., 2010). Similarly, In preclinical models, oral administration of Bifidobacterium animalis subsp. lactis MN-Gup (1×10^10^ CFU/kg/day via gavage) for 6 weeks in high-fat diet/streptozotocin-induced type 2 diabetic mice significantly attenuated fasting blood glucose levels, suggesting insulin sensitivity (reduced HOMA-IR), and enhanced endogenous GLP-1 secretion and short-chain fatty acid (acetate) production, alongside favorable restoration of gut microbiota composition (Zhang et al., 2024). Prebiotics are non-digestible food components that selectively stimulate the growth or activity of beneficial gut microorganisms, acting as fuel for the microbiome (Gibson et al., 2017). Inulin, fructo-oligosaccharides (FOS), and galactooligosaccharides (GOS) represent the most commonly studied prebiotics in metabolic disease (Wilson and Whelan, 2017). By providing specific substrates that human enzymes cannot break down, prebiotics encourage the proliferation of beneficial bacteria such as Bifidobacterium and Lactobacillus.

Prebiotic supplementation consistently increases fecal abundance of Bifidobacterium and often elevates SCFA production, particularly butyrate and propionate (Toe et al., 2020). These metabolites play crucial roles in regulating appetite and glucose metabolism. Studies appear to demonstrate that improvements in glucose homeostasis, satiety hormones like GLP-1 and PYY, and inflammatory markers occur with prebiotic consumption, though effects on body weight are typically modest (Rabiei et al., 2019; Irfan et al., 2026). This suggests that prebiotics primarily improve metabolic health through mechanisms other than simple weight loss, such as enhancing insulin sensitivity and reducing systemic inflammation.

Akkermansia muciniphila, a mucin-degrading bacterium residing in the intestinal mucus layer, has emerged as a particularly promising therapeutic candidate, often referred to as a next-generation probiotic (Cani et al., 2022). Reduced abundance of A. muciniphila is consistently observed in obesity and type 2 diabetes, correlating with increased intestinal permeability (Zhang et al., 2025). Supplementation with live or pasteurized A. muciniphila has demonstrated remarkable efficacy in preclinical models, improving glucose tolerance, reducing adiposity, and enhancing intestinal barrier function (Luo et al., 2022).The fact that pasteurized forms are effective suggests that bacterial components, such as outer membrane proteins, may mediate some of these benefits.

Recent human trials support the therapeutic potential of A. muciniphila supplementation. A landmark study demonstrated that pasteurized A. muciniphila administration improved insulin sensitivity, reduced inflammatory markers, and decreased body weight in overweight/obese individuals (Depommier et al., 2019). Notably, efficacy appears dependent on baseline A. muciniphila levels, suggesting a personalized medicine approach may be optimal (Zhang et al., 2025). Individuals with very low baseline levels may benefit most, while those with higher levels might see diminishing returns.

Given the critical role of butyrate in maintaining intestinal health and regulating inflammation, direct supplementation with butyrate-producing bacteria represents an attractive therapeutic strategy (Rivière et al., 2016). Species within the families Ruminococcaceae and Lachnospiraceae, particularly Faecalibacterium prausnitzii, are principal butyrate producers in the human colon (Louis and Flint, 2017; Martín et al., 2023). prausnitzii is considered a key indicator of gut health due to its anti-inflammatory properties.

F. prausnitzii exhibits potent anti-inflammatory properties and is consistently reduced in inflammatory bowel disease and metabolic syndrome (He et al., 2021). Preliminary studies of F. prausnitzii supplementation show promise for improving intestinal barrier function and reducing systemic inflammation, although technical challenges related to its strict anaerobic nature have hindered widespread clinical application (Wang X. et al., 2024). Advances in encapsulation technology and formulation are currently addressing these stability issues.

Advances in synthetic biology enable the engineering of probiotic strains with enhanced therapeutic capabilities, opening new frontiers in treatment (Li et al., 2025). Engineered strains can be designed to produce specific metabolites, secrete therapeutic peptides, or sense and respond to pathological conditions in the gut environment (Riglar and Silver, 2018). For example, bacteria could be engineered to release GLP-1 in response to high glucose levels. While still largely in preclinical development, engineered probiotics represent a promising frontier in precision microbial therapeutics (Wang et al., 2024a).

Finally, diet represents the most powerful and accessible tool for modulating the gut microbiome. The Western diet, characterized by high fat, high sugar, and low fiber content, promotes dysbiosis and metabolic inflammation (Martinez et al., 2017). Conversely, Mediterranean-style diets rich in fiber, polyphenols, and unsaturated fats support microbial diversity and beneficial metabolite production (Nagpal et al., 2019; Lauria et al., 2025). Dietary fiber, in particular, serves as the primary substrate for SCFA production. High-fiber diets increase fecal butyrate and propionate levels, improve intestinal barrier function, and reduce systemic inflammation (Liu et al., 2022). Fermentable fibers including resistant starch, inulin, and various oligosaccharides selectively stimulate beneficial microbial populations, reinforcing the concept that long-term metabolic health is deeply rooted in daily dietary choices (Rezende et al., 2021). Overall, while preclinical animal models provide foundational mechanistic insights into microbiome-driven metabolic dysfunction, translating these findings to human clinical practice reveals a more nuanced picture. Human evidence—derived from prospective cohort studies, systematic reviews, and randomized controlled trial (RCTs) evaluating FMT, Akkermansia muciniphila, and dietary patterns like the Mediterranean diet—demonstrates that clinical efficacy is frequently modest and highly contingent upon baseline host-microbiome interactions and lifestyle factors. Consequently, human trials underscore that microbiome-targeted interventions achieve maximum therapeutic efficacy not as isolated remedies, but as personalized adjuncts to lifestyle modifications. A summary of these and more recent studies is provided in **Table 2**.

**Table 2:** Overview of microbial restructuring strategies and their clinical/preclinical impact on metabolic inflammation.

Intervention	Target/agent	Primary mechanism	Key metabolic outcome	Ref
Fecal Microbiota Transplantation (Double-Blind RCT, Human)	Lean donor allogenic microbiota	Ecosystem restructuring & enrichment of butyrate-producing bacteria	Significant increase in insulin sensitivity (rate of glucose disappearance: 26.2 to 45.3 μmol/kg/min)	(Vrieze et al., 2012)
Fecal Microbiota Transplantation Double-Blind RCT (Human)	Lean donor microbiota	Global ecosystem reset; restoration of SCFA-producers	Transient improvement in insulin sensitivity	(Kootte et al., 2017)
Probiotics RCT	L. gasseri SBT2055	Competitive exclusion of pathogens; SCFA modulation	Significant reduction in visceral fat area (−4.6%), subcutaneous fat (−3.3%), body weight (−1.4%/−1.1 kg), BMI (−1.5%), and waist circumference (−1.8%/−1.7 cm)	(Kadooka et al., 2010)
Prebiotics Preclinical Model	Bifidobacterium spp	Selective fermentation; stimulation of Bifidobacterium	Improved GLP-1 secretion and reduced systemic LPS	(Cani et al., 2009)
Next-Gen Probiotics RCT	Akkermansia muciniphila (ATCC BAA-835)	Outer membrane protein (Amuc_1100) TLR2 activation, mucosal barrier reinforcement, and gut permeability reduction	Improved insulin sensitivity (+28.6%), decreased insulinemia (−34.1%), reduced total cholesterol (−8.7%), modest weight loss (−2.27 kg), and lowered liver injury markers	(Depommier et al., 2019)
Butyrate Producers Experimental inflammation model	Faecalibacterium prausnitzii	Butyrate-mediated HDAC1 inhibition, suppression of the IL-6/STAT3/IL-17 pathway, and upregulation of Foxp3 expression	Suppression of IL-6/STAT3/IL-17 pathway; ↑ Foxp3 expression & Restored Th17/Treg balance; significant reduction in colitis/systemic inflammation	(Zhou et al., 2018)
Engineered Probiotics Preclinical/Animal Model	E. coli Nissle 1917 (EcN) expressing GLP-1 with lipid membrane coating (LEG system)	Targeted pancreatic biochemotaxis, continuous local GLP-1 secretion, protection of islet β -cells via immune regulation, and gut microbiota modulation	Long-term blood glucose control, restoration of islet β -cell function, increased microbial richness/diversity, and high biosafety with alternate-day oral dosing	(Wang et al., 2024b)
Dietary Modulation (RCT - 6-Year Follow-up)	Mediterranean Diet (supplemented with extra virgin olive oil)	SCFA production, enhancement of barrier integrity, and reduction of hepatic lipid accumulation	Significant reduction in Fatty Liver Index (FLI), suppression of waist circumference growth (−0.51 cm/year), and lower BMI trajectory over 6 years	(Cueto-Galan et al., 2017)
Fecal Microbiota Transplantation Phase 2 RCT	Lean donor microbiota	Broad restructuring of the gut metagenome, restoration of short-chain fatty acid/amino acid metabolites (e.g., ↓ serum kynurenine and branched-chain amino acids), and expansion of beneficial taxa (Bacteroides, Roseburia, Bifidobacterium)	Favorable shifts in gut microbiome and serum/fecal metabolite networks, improved LDL cholesterol and appetite scores, but no significant changes in HOMA-IR or total body weight	(Ghorbani et al., 2023)
Probiotics RCT	Multi-strain consortium	Restoration of intestinal barrier integrity; ↓ paracellular permeability	Significant improvement in intestinal barrier function (up to 48% reduction in permeability), increased HDL-cholesterol, and improved obesity-related anthropometric indices	(Chaiyasut et al., 2022)

BCAAs, branched-chain amino acids; BMI, body mass index; EcN, Escherichia coli Nissle 1917; FLI, fatty liver index; FMT, fecal microbiota transplantation; GLP-1/2, glucagon-like peptide-1/2; HDAC1, histone deacetylase 1; HDL, high-density lipoprotein; HOMA-IR, homeostatic model assessment of insulin resistance; LDL, low-density lipoprotein; LPS, lipopolysaccharide; PREDIMED, Prevención con Dieta Mediterránea; RCT, randomized controlled trial; SCFA, short-chain fatty acid; Th17, T helper 17 cells; TLR2/4, Toll-like receptor 2/4; Treg, regulatory T cells; ZO-1, zonula occludens-1.

## 5 Integration and future directions

The substantial inter-individual variability in gut microbiota composition suggests that optimal therapeutic strategies may vary significantly across individuals, marking a pivotal shift away from one-size-fits-all medical approaches toward precision medicine (Montazeri-Najafabady, 2025). This heterogeneity is not merely noise in the data but reflects deep biological differences shaped by a lifetime of unique environmental exposures. Factors including baseline microbiome composition, host genetics, long-term dietary patterns, age, and medication use all influence treatment responses, creating a complex matrix of variables that determine whether a specific intervention will succeed or fail (Rothschild et al., 2018). For instance, an individual’s genetic background can influence the immune system’s response to microbial components, while prior antibiotic use may leave ecological niches open for either beneficial engraftment or pathogenic colonization. Consequently, two individuals with identical body mass indices may respond differently to the same probiotic strain due to these underlying contextual factors.

The development of microbiome-based biomarkers to predict therapeutic response represents an active and critical area of research (Meade et al., 2023). Scientists are increasingly leveraging machine learning algorithms and multi-omics data integration to identify specific microbial signatures that correlate with positive metabolic outcomes. These predictive models aim to stratify patients into responder and non-responder groups before therapy initiation, thereby optimizing clinical trial designs and improving patient care (Thakur, 2025). By identifying key taxa or functional genes associated with successful weight loss or improved insulin sensitivity, clinicians could tailor interventions to the individual’s unique microbial landscape. This approach moves beyond simple taxonomic identification to functional profiling, assessing what the microbiome is doing rather than just who is there. Such biomarkers could include the abundance of specific short-chain fatty acid producers, the ratio of Firmicutes to Bacteroidetes, or the presence of bile acid-modifying enzymes (Shin et al., 2025).

Given the complexity of obesity-associated dysbiosis, which involves multiple interconnected pathways of inflammation, energy harvest, and barrier dysfunction, combination approaches may prove more effective than single interventions (Singh et al., 2023). Monotherapies often fail to address the multifaceted nature of the disease, whereas synergistic strategies can target multiple mechanisms simultaneously. Pairing prebiotics with specific probiotic strains, known as synbiotics, ensures that the introduced beneficial bacteria have the necessary substrates to survive and thrive in the hostile environment of the obese gut (Boyajian et al., 2024). This synergy enhances the persistence and activity of the probiotic strains, leading to more robust metabolic improvements. Furthermore, combining microbial therapies with dietary modification addresses both the supply side (microbial input) and the demand side (host substrate availability). Dietary changes provide the long-term environmental stability needed for microbial interventions to take root, preventing the reversion to baseline dysbiosis that often plagues standalone treatments (Leeming et al., 2019).

Another promising strategy involves sequencing interventions to first deplete pathogenic populations followed by beneficial colonization. This “clear and replace” approach mimics ecological succession, where removing competitive inhibitors allows newly introduced beneficial species to establish themselves more effectively. For example, using targeted antimicrobials or bacteriophages to reduce pro-inflammatory Enterobacteriaceae could create ecological space for butyrate-producing bacteria to engraft following FMT or probiotic administration (Schupack et al., 2022). This temporal dimension of therapy adds a layer of sophistication to treatment protocols, recognizing that the order of interventions matters as much as the interventions themselves.

While microbiome-targeted therapies generally demonstrate favorable safety profiles, potential risks warrant careful consideration and rigorous monitoring (Metris et al., 2025). The field is still young, and long-term safety data are limited, particularly for next-generation live biotherapeutic products. FMT carries inherent risks of pathogen transmission, including viruses, parasites, and multidrug-resistant bacteria, though rigorous donor screening and advanced molecular testing significantly mitigate this concern (Baxter et al., 2015). Recent cases of severe infections following FMT have highlighted the need for standardized safety protocols and regulatory oversight. Additionally, there is the theoretical risk of transferring undesirable traits, such as susceptibility to metabolic disease or autoimmune conditions, although current evidence suggests this risk is low when donors are carefully selected based on metabolic health.

Probiotic administration, while generally safe for healthy individuals, may pose risks in immunocompromised individuals, those with central venous catheters, or patients with short bowel syndrome (Doron and Snydman, 2015). In these vulnerable populations, live bacteria can potentially translocate across the intestinal barrier, leading to bacteremia or fungemia. Furthermore, the unintended metabolic consequences of microbial manipulation require careful monitoring (Zheng et al., 2026). Altering the gut microbiome can have downstream effects on drug metabolism, neurotransmitter production, and immune function that are not yet fully understood. For instance, changes in microbial bile acid metabolism could influence the efficacy of certain medications or contribute to gallstone formation (Tan et al., 2025). There is also the concern that excessive modulation of the microbiome could disrupt established ecological balances, leading to new forms of dysbiosis. Therefore, personalized monitoring, including regular assessment of inflammatory markers and metabolic parameters, is essential to ensure that therapeutic benefits outweigh potential risks. As the field matures, the establishment of comprehensive registries and long-term follow-up studies will be crucial to defining the safety landscape of these innovative therapies. Despite these promising avenues, several overarching limitations must be acknowledged when evaluating current evidence. Inter-individual heterogeneity in obesity phenotypes, along with unmeasured confounding from background diets and concomitant medications (such as metformin), significantly influences intervention outcomes. Furthermore, technical variations across microbiome sequencing protocols, together with the heavy reliance on fecal sampling rather than mucosal-associated microbiota, limit cross-study comparability and anatomical precision. The clinical translation of these therapies is further constrained by a scarcity of long-term trials, potential publication bias favoring positive results, and the inherent biological friction in extrapolating preclinical rodent findings to complex human biology. Addressing these methodological and biological hurdles will be essential to establish durable, reproducible biotherapeutics.

## 6 Conclusion

The gut microbiota-immune-metabolic axis has emerged as a key mediator in modern metabolic research, providing valuable insights into how chronic low-grade inflammation contributes to the onset and progression of obesity. Rather than acting as passive observers, dysbiotic microbial communities actively perpetuate meta-inflammation via systemic LPS translocation, compromised intestinal barrier integrity, and depleted short-chain fatty acid production. Re-establishing ecosystem stability through microbiota-targeted interventions—ranging from traditional probiotics and FMT to next-generation biotherapeutics—offers a transformative approach that targets the biological drivers of metabolic dysfunction rather than merely managing downstream clinical symptoms.

However, moving from promising proof-of-concept studies to routine clinical adoption requires overcoming critical translational and mechanistic barriers. The future success of microbiome medicine hinges on transitioning from descriptive, taxon-based correlations to causal, multi-omics-driven frameworks. Crucially, addressing the challenge of transient efficacy and inter-individual response variability demands standardized protocols that prioritize long-term microbial strain engraftment, functional metabolic output (such as SCFA and bile acid signaling), and stringent safety profiles for engineered bio-therapeutics.

Ultimately, realizing the full translational potential of microbial restoration necessitates a paradigm shift toward precision metabolic care. By integrating multi-omics biomarkers, patient-specific baseline profiles, and synergistic combination therapies, clinicians can move beyond empirical trial-and-error interventions. As continuous research bridges fundamental mechanistic insights with prospective human clinical trials, microbiota-targeted therapeutics hold immense promise to refine risk stratification, prevent disease progression, and reshape the landscape of personalized metabolic healthcare.
